# Recent Development of Dual-Dictionary Learning Approach in Medical Image Analysis and Reconstruction

**DOI:** 10.1155/2015/152693

**Published:** 2015-05-18

**Authors:** Bigong Wang, Liang Li

**Affiliations:** ^1^Department of Engineering Physics, Tsinghua University, Beijing 100084, China; ^2^Key Laboratory of Particle & Radiation Imaging (Tsinghua University), Ministry of Education, Beijing 100084, China

## Abstract

As an implementation of compressive sensing (CS), dual-dictionary learning (DDL) method provides an ideal access to restore signals of two related dictionaries and sparse representation. It has been proven that this method performs well in medical image reconstruction with highly undersampled data, especially for multimodality imaging like CT-MRI hybrid reconstruction. Because of its outstanding strength, short signal acquisition time, and low radiation dose, DDL has allured a broad interest in both academic and industrial fields. Here in this review article, we summarize DDL's development history, conclude the latest advance, and also discuss its role in the future directions and potential applications in medical imaging. Meanwhile, this paper points out that DDL is still in the initial stage, and it is necessary to make further studies to improve this method, especially in dictionary training.

## 1. Introduction

Compressive sensing (CS) is a novel theory in information acquisition and processing [1]. Since general signals are broadband, traditional signal reconstruction methods usually adopt Nyquist Sampling, requiring high sample rate and long processing time. However, CS theory offers a way to restore signal accurately with less measurement by solving an optimization problem in which signal is sparse, represented using a basis matrix, and the high-dimensional transformation is projected to a lower dimensional subspace. Therefore, CS theory has been widely recognized and applied in various fields.

Some groups focus on studies of CS applications and have developed various braches such as Bayesian CS and 1-Bit CS [2–4]. After it is applied in medical imaging reconstruction, CS theory is proven to be a method that effectively retains high image quality using undersampling measurement data in different imaging modalities including computed tomography (CT) and magnetic resonance imaging (MRI) [5–7]. Besides, CS theory shows great potential in multimodalities image reconstruction, one of the future directions of medical imaging.

Dictionary learning (DL) is a typical method of CS image reconstruction. In this method, sampled data is compressible in specific transform domain, and transformation coefficients are projected to a lower dimensional vector with essential image information retained well. As a result, complex reconstruction problem is simplified to an optimization problem. Usually, one should take three problems into consideration to solve image reconstruction problems using DL methods. First, design an overcompleted dictionary which can represent a signal sparsely. Second, get a measurement matrix strictly satisfied with isometry property. Third, develop a fast signal reconstruction algorithm with good robustness. The designed dictionary is important to the accuracy of CS image reconstruction. In DL method, the dictionary is self-adaptive and flexible; it is trained by particular image samples or group of images. Using different training methods, the image sparseness is quite different [8].

Though DL-based approach has been recognized in medical image reconstruction field, single dictionary applied in the whole image process brings out a limit in image quality. That means only one dictionary is far from enough as the prior information. In order to improve image quality, research scholars have optimized DL method to dual-dictionary learning (DDL) which has more diverse prior information in imaging modalities like CT and MRI. DDL method was initially developed for image super-resolution. Lu et al. [9, 10] applied this method for CT reconstruction. Song et al. [11] used it in 3D MRI reconstruction. DDL shows a great potential in medical image reconstruction.

In this paper, we discuss the DL method in Section 2. Based on DL method, we review DDL's history and new development in Section 3, including its theory, feasibility demonstration, and the application in different fields. In Section 4, we discuss the use of DDL in medical image analysis. In the section of Discussion and Conclusion, we summarize algorithms and explore the future directions in medical image reconstruction.

## 2. Dictionary Learning (DL) Algorithm

### 2.1. DL Method and Theory

According to the CS theory, an undersampling image reconstruction problem is to solve an underdetermined system of linear equations *F*_*u*_ *x* = *y* by minimizing the *l*_0_ quasi norm (e.g., number of nonzeros) of the sparsified transform Ψ*x*; it means the image *x* is sparse after a completed sparse transform Ψ ∈ *ℜ* ^*M*×*N*^. The corresponding optimization problem is

(1) $$\begin{array}{c} \underset{x}{\min}{\left\|\Psi x\right\|}_{0}\;\text{s}.\text{t}.\;\;F_{u}x=y. \end{array}$$

In (1), *x* is the image to be reconstructed, *F*_*u*_ is the codebook for the given measurements *y*. Equation (1) is also known as a sparse coding problem, which is a NP-hard problem (nondeterministic polynomial). It can be solved by some greedy algorithms, for example, orthogonal matching pursuit (OMP) [12]. It is notable that if the *l*_0_ norm is replaced with *l*_1_ norm, the problem can be solved by linear programming in the real domain or second order cone programming in the complex domain.

Given an image *X* of size *N* × *N*, it can be decomposed into some small patches of size *b* × *b*, *b* ≪ *N*. Each patch can be expressed as a *n* = *b* ^2^ dimensional vector **x** ∈ *ℜ* ^*n*^. All the patches are extracted from the object image *X* according to the patch size and the slide distance. A dictionary *D* ∈ *ℜ* ^*n*×*K*^ is a matrix that consists of *K* atoms *d*_*k*_ ∈ *ℜ* ^*n*=*b*×*b*^ which are the columns of the dictionary. As *d*_*k*_ is the patch vector from sample images, the initial dictionary constructed from the extracted patches is usually redundant or overcompleted; that is, *N* ≪ *K*. Using specific atoms of initial dictionary *D*, each vector **x** in the image can be approximately represented as sparse coefficient [13]. Consider

(2) $$\begin{array}{c} {\left\|x-D\alpha \right\|}_{2}^{2}<\varepsilon , \end{array}$$

where *ε* > 0 for the error bound and **α** ∈ *ℜ* ^*K*^ for the sparse representation vector which has few nonzero elements: ‖**α**‖_0_ ≪ *N* ≪ *K*, *i* = 1,2,…, *K*. To get the sparse representation of the vector **x**, one can minimize the *l*_0_ norm as

(3) $$\begin{array}{c} \underset{\alpha }{\min}{\left\|\alpha \right\|}_{0}\;\text{s}.\text{t}.\;\;{\left\|x-D\alpha \right\|}_{2}^{2}<\varepsilon . \end{array}$$

If an image contains *S* patches, DL is to find a dictionary $\tilde{D}$ in which all the patches should be sparsely represented as follows:

(4) $$\begin{array}{c} \underset{D,\alpha }{\min}\overset{S}{\underset{s=1}{\sum }}\left({\left\|x_{s}-\tilde{D}\alpha_{s}\right\|}_{2}^{2}+\nu {\left\|\alpha_{s}\right\|}_{0}\right). \end{array}$$

Usually, if *ν* is fixed by specific value, (3) is equivalent to solve the following problem:

(5) $$\begin{array}{c} \underset{D,\alpha }{\min}\overset{S}{\underset{s=1}{\sum }}\left({\left\|x_{s}-\tilde{D}\alpha_{s}\right\|}_{2}^{2}\right)\;\text{s}.\text{t}.\;\;{\left\|\alpha_{s}\right\|}_{0}<T_{0}. \end{array}$$

### 2.2. Dictionary Construction

DL problem is NP-hard because it turns to a sparse coding problem when *D* and **x** are fixed. Currently, mainly four adaptive dictionary training algorithms were proposed to solve such a dictionary learning problem.

1. Direct method (DM): DM is an original method that preserves all the details in the sample images because of a direct extraction process, and then a target image can be fully recovered as the patches are well chosen. Usually, this method is effective in super-resolution image reconstruction.
2. Method of optimal directions (MOD): MOD fixes the coefficients corresponding to the dictionary vectors and then updates the atoms by minimizing the residuals between the training vectors and its representations. The main advantage of MOD is that it gives the optimal adjustment of the dictionary vectors in each iteration. Usually, it provides better convergence properties in ECG (electrocardiogram) signals [14].
3. Generalized principal component analysis (GPCA): GPCA is a general method for modeling and segmenting some mixed data using a collection of subspaces. By introducing certain algebraic models and techniques into data clustering, traditionally a statistical problem, GPCA offers a new spectrum of algorithms for data modeling and clustering [15].
4. *K*-means singular value decomposition (*K*-SVD): *K*-SVD is an iterative method updating the dictionary atoms to fit the data better. The method does SVD on the errors and updates the current dictionary atom and coefficient simultaneously with the item which has the minimum error. As the most widely used method to train the dictionary, *K*-SVD has an excellent convergence and sparsity [16].

**Figure 1:**
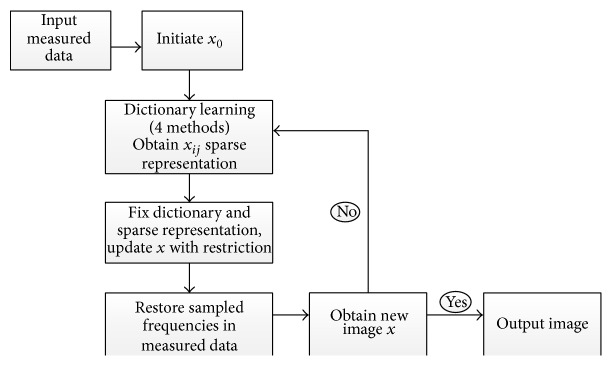
The algorithm block diagram of diction learning applied in image reconstruction.

Dictionary learning can be used to reconstruct image; a classic algorithm is summarized in Figure 1. Given an initial value *x*_0_ (initial dictionary), do dictionary learning using appropriate training method and obtain the sparse representation, and then update *x* under specific transform (i.e., wavelet, Fourier) and output the result after several iterations at last.

## 3. DDL Algorithm in Image Analysis

### 3.1. From Single to Dual-Dictionary

DL method is widely used in image restoration [17–19], super-resolution reconstruction [20–23], image deblurring [24–26], denoising [27–32], medical image reconstruction [13, 33], image prediction [34], and image inpainting [35]. However, both dynamitic atoms in each iteration step and certain noise in measurement data would increase iteration time making DL method slow in most cases. As to improve DL's inefficiency, some come up with the solution that by introducing two or more dictionaries image quality would be further improved within less time. One of the improved methods is dual-dictionary learning (DDL).

DDL theory is first introduced by Curzion et al. as PADDL; it aimed to train a linear mapping in the case of a single dictionary. Note that this method is not using two different dictionaries but training one dictionary with its “dual” dictionary. In PADDL method, the essential concept is to update the dictionary *D* = [*d*_1_,…, *d*_*K*_] ∈ *ℜ* ^*d*×*K*^ by means of its “dual” dictionary *C* = [*c*_1_,…,*c*_*K*_]^*T*^ ∈ *ℜ* ^*K*×*d*^, as an auxiliary item. It aims to find an optimal pair of linear operators *D* by minimizing the following:

(6) ED,C,U=X−DUF2+ηU−CXF2+τU1s.t.  di2,ci2≤1,

where *X* ∈ *ℜ* ^*d*×*N*^ is the matrix to be trained and *U* ∈ *ℜ* ^*K*×*N*^ is the representation. The *c*_*i*_ can be treated as filters to approximate its optimal *u*. *η* is the weight parameters.

The result shows that this dual-dictionary training method can be applied well in calculating the sparse representations [36].

### 3.2. DDL in Super-Resolution Reconstruction

Zhang et al. proposed an efficient sparse representation method to solve image super-resolution reconstruction via DDL [37]. In this work, they assume that image patches with different resolution can share the same underlying sparse representation. Thus, given a dictionary pair {*D*_*h*_, *D*_*l*_}, where *h* stands for high resolution and *l* stands for low resolution, the sparse representation of *x*_*i*_ from low-resolution image *X*_*l*_ is similar as (3). Consider

(7) $$\begin{array}{c} {\hat{z}}_{i}=\arg\;\min{\left\|z_{i}\right\|}_{1}\;\text{s}.\text{t}.\;\;{\left\|D_{l}z_{i}-x_{i}\right\|}_{2}^{2}\leq \varepsilon . \end{array}$$

With the sparse representation vector *z*_*i*_, the high-resolution patch can be approximately expressed as *x*_*i*_′ = *D*_*h*_ *z*_*i*_. Put all the high-resolution patches back into corresponding positions and perform normalization. Finally we obtain the estimation of the high-resolution image *x*′.

The optimization model for learning coupled dictionaries with “dual” is as follows:

(8) Dh,Dl,Cl,Z =argmin⁡Dh,Dl,Cl,ZXc−DcZF2+ηZ−ClXlF2+λZ1     s.t.  Di22,Ci22≤1.

$X_{c}={\left[(1/\sqrt{M}){X_{h}}^{T},(1/\sqrt{N}){X_{l}}^{T}\right]}^{T}\in {ℜ}^{(M+N)\times L}$, in which *M* and *N* are the dimension of the high- and low-resolution patches. $D_{c}={\left[(1/\sqrt{M}){D_{h}}^{T},(1/\sqrt{N}){D_{l}}^{T}\right]}^{T}\in {ℜ}^{(M+N)\times K}$. *C*_*l*_ ∈ *ℜ* ^*K*×*N*^ is the dual of *D*_*l*_ as mentioned in Section 2.1. After multiplying *z*_*i*_ = *C*_*l*_ *x*_*i*_ by *D*_*h*_, we acquire the high-resolution patch *x*_*i*_′. In this method, *D*_*l*_ and *D*_*h*_ are treated as one dictionary and trained simultaneously with their dual, which refers to *D*_*l*_ and *D*_*h*_.

With the approximate sparse coding procedure via model (8), the result shows that their method speeds up the overall super-resolution process significantly.

### 3.3. DDL in Image Restoration

Similar to HaiChaos' work, Wang et al. also applied DDL in image restoration [38]. They solved the problem of restoring the lost part of high-frequency detail information of images.

Wang et al. reconstructed the high-frequency (HF) details from the low-resolution images using the prior models. HF is decomposed into a combination of two components, main high-frequency (MHF) and residual high-frequency (RHF). Wang et al. restored MHF and RHF, respectively, with dual-dictionary and then added up MHF and RHF at last. For dictionary construction, *K*-SVD was used to train the two dictionaries. The experiment result reveals that the PSNR values are better than bicubic and sparse representation algorithm.

### 3.4. DDL in Human Pose Estimation

Ji and Su proposed a new method for robust 3D human pose estimation using DDL [39]. In their study, they constructed two dictionaries simultaneously including visual observation dictionary and body configuration dictionary. Both of the two dictionaries share with a same sparse representation with respect to every visual observation and its corresponding 3D body pose.

Since outline features are usually corrupted, the optimization model for robust human pose estimation is as follows:

(9) min⁡A,B,E,RE1+λR1s.t. X=AR+E   Y=BR,

where *X* ∈ *ℜ* ^*m*×*n*^ for observation data matrix, *A* ∈ *ℜ* ^*m*×*d*^ for observation dictionary, *Y* ∈ *ℜ* ^*k*×*n*^ for 3D pose data matrix, and *B* ∈ *ℜ* ^*k*×*d*^ for body configuration dictionary. *R* ∈ *ℜ* ^*d*×*n*^ for common sparse representation of *X* and *Y*, and *E* is the corruption item to be minimized.

To solve problem (9), Hao and Fei used an inexact Augmented Lagrange Multiplier (IALM) method to update the two dictionaries. More details related to the IALM method can be learned from [29].

The experimental results show that their approach performs well in recovering outlines from corrupted data compared with other methods.

## 4. DDL Algorithm in Medical Image Reconstruction

Recently, DDL has gained attention in medical image reconstruction, which can improve image qualities and accelerate reconstruction process.

### 4.1. Method and Theory

Let *u* ^*l*^ be a low-quality image and *D*_*l*_ = [*d*_1_^*l*^, *d*_2_^*l*^,…, *d*_*K*_^*l*^], and let *D*_*l*_ ∈ *ℜ* ^*n*×*K*^ be a low dictionary constructed from *u* ^*l*^. Similarly, let *u* ^*h*^ be the high-quality counterpart of *u* ^*l*^ and *D*_*h*_ = [*d*_1_^*h*^, *d*_2_^*h*^,…, *d*_*K*_^*h*^]; *D*_*h*_ ∈ *ℜ* ^*n*×*K*^ constructed from *u* ^*h*^. As a corresponding relation between *u* ^*l*^ and *u* ^*h*^, they can be connected with a general following model:

(10) $$\begin{array}{c} u^{l}=Qu^{h}+\varepsilon^{l}, \end{array}$$

where *ε* ^*l*^ is the noise and *Q* is the transform operator. For a specific *u* ^*h*^, we can assume that each patch *u*_*i*_^*h*^ in *u* ^*h*^ can be expressed as the linear combination of the atoms in the following dictionary *D*_*h*_:

(11) $$\begin{array}{c} u_{i}^{h}=D_{h}\alpha_{i}+\eta , \end{array}$$

where *η* is the error; ‖*η*‖_2_^2^ < *ε*. **α**_*i*_ is sparse coefficient, ‖**α**_*i*_‖_0_ ≪ *K*. Combining (11) and (10) gives

(12) $$\begin{array}{c} {\left\|u_{i}^{l}-D_{l}\alpha_{i}\right\|}_{2}^{2}<\delta ={\left\|u_{i}^{l}-QD_{h}\alpha_{i}\right\|}_{2}^{2}<\delta . \end{array}$$

According to the above derivations which are referred to as the Sparse-Land Model, the low-quality patch *u*_*i*_^*l*^ can be sparse coded by the same vector **α**_*i*_ under dictionary *D*_*l*_ = *QD*_*h*_. Thus, given the dictionaries *D*_*l*_ and *D*_*h*_ with accurate one-to-one mapping atoms, we can approximately recover *u*_*i*_^*h*^ simply by multiplying *D*_*h*_ and the sparse representation obtained from *D*_*l*_ as follows:

(13) $$\begin{array}{c} u_{i}^{h}=D_{h}\alpha_{i}+\varepsilon_{i}. \end{array}$$

**Figure 2:**
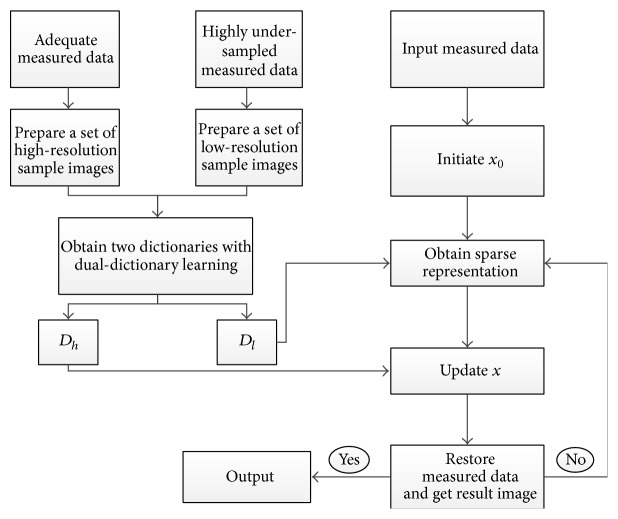
The general workflow for DDL method.

The general workflow for DDL method in medical image reconstruction is summarized in Figure 2. Given two sets of measured data (high-resolution sample images and low-resolution sample images), we can obtain two dictionaries *D*_*l*_ and *D*_*h*_ using appropriate training methods (DM, MOD, GPCA, or *K*-SVD). When a measured data is input, we can obtain the sparse representation with *D*_*l*_ and then update the *x* using *D*_*h*_.

### 4.2. DDL in CT Reconstruction

Computed tomography (CT) reconstruction is a process obtaining the tomographic image of human body from X-ray projection data. The reconstruction methods can be divided into two types, analytic and iterative methods. In recent years, CS-based iterative method was applied in 3D X-ray image reconstruction. It performs more flexible and accurate than analytic method in most of cases. Some typical topics include interior CT problem, low-dose imaging, and incomplete data reconstruction [40–44].

Lu et al. made a progress in few-view image reconstruction of CT images (SART-TV-DL) [9, 10] using DDL. Since each pair of corresponding sample images is reconstructed from the same object just different in view numbers of projection, a high-quality image and its low-quality counterpart have the relationship described in (10).

In their work, a set of high-quality images which were reconstructed with SART algorithm from adequate projection were used to construct a high-quality dictionary *D*_*h*_; however, according to the pixel-to-pixel mapping rule, a low-quality dictionary *D*_*l*_ can be also generated from a set of blurry images which were reconstructed from under-sampled projection data. To solve the dictionary training problem, they used DM mentioned in Section 1 because it could reserve most details of the sample images. Moreover, this method can generate dictionaries easiest and fastest.

However, in a CT image, pixel values alone cannot reflect the relationship of the adjacent two pixels. Therefore, in addition to DM, they used pixel values combined with its first-order gradient vector along *x* and *y* direction to provide more information of an image vector for each patch. That is, if an image patch is of size $\sqrt{n}\times \sqrt{n}$, the atom in the dictionary had 3*n* features because of the gradient. As the dictionaries were redundant or overcomplete, they reduced the redundancy of the dictionaries by means of setting a minimum Euclidean distances threshold.

The real data results demonstrate the potential of SART-TV-DL algorithm in CT image reconstruction with 30–50 views. It contributes to some preclinical and clinical applications such as C-arm, breast CT, and tomosynthesis.

Different from Lu's work, Cao and Xing applied DDL in CT limited angle reconstruction [45]. In his work, a two-dictionary learning (ART-TV-TDL) algorithm is proposed to remove the limited angle artifacts. The two dictionaries were, respectively, object dictionary *D*_*o*_ learned from a high-quality training image and artifact dictionary *D*_*a*_ from artifact image. A limited angle reconstruction $\tilde{X}$, which could be divided into the object part *X*_*o*_ and the artifact part *X*_*a*_, had the different sparse representation coefficients with *D*_*o*_ and *D*_*a*_ as follows:

(14) min⁡αoX~−Doα22 s.t.  αi0≤L1,min⁡αaX~−Daβ22 s.t.  βi0≤L2.

Here *α* and *β* are the sparse coefficient with *L*_1_ and *L*_2_ sparsity; the training method was *K*-SVD in this work. To get a better image with restrain artifacts, they combined these two representations for iterative reconstruction. Consider

(15) $$\begin{array}{c} X^{\text{next}}=\lambda_{o}D_{o}\alpha -\lambda_{a}D_{a}\beta +\lambda X^{\text{current}}, \end{array}$$

where *λ*_*o*_, *λ*_*a*_, and *λ* are parameters to balance the effect. Their results show that the ART-TV-TDL method has smaller RMSE values in different limited angles (90 and 120) compared with ART-TV method.

### 4.3. DDL in 3D MRI Reconstruction

Song et al. proposed a novel method for multislice (3D) MRI reconstruction from undersampled *k*-space data using dual-dictionary learning (Dual-DL-MRI) [11].

For a high-resolution *M* × *N* × *H* MRI images series *S*_*h*_, one can represent them as one vector *s*_high_ ∈ *ℜ* ^*MNH*×1^ of length *MHN* and get its undersampled *k*-space measurements *y* by Fourier transform $y={\tilde{F}}_{u}s_{\text{high}}$. ${\tilde{F}}_{u}$ is a three-dimension undersampling Fourier matrix. Therefore, the corresponding series *s*_low_ ∈ *ℜ* ^*MNH*×1^ can be reconstructed from undersampled *k*-space by inverse Fourier transform as follows:

(16) $$\begin{array}{c} s_{\text{low}}={\tilde{F}}^{*}y={\tilde{F}}^{*}{\tilde{F}}_{u}s_{\text{high}}=Qs_{\text{high}}. \end{array}$$

As we can see, (16) is one form of (10), which demonstrates the possibility of dual-dictionary in MRI reconstruction.

To construct dual-dictionary, they used *K*-SVD method to train the two dictionaries simultaneously to ensure the matching accuracy (one-to-one correspondence); *D*_*l*_ and *D*_*h*_ can be obtained by

(17) $$\begin{array}{c} \underset{D,\alpha_{i}}{\min}\underset{i}{\sum }{\left\|\alpha_{i}\right\|}_{0}\;\text{s}.\text{t}.\;\;{\left\|s_{i}-D\alpha_{i}\right\|}_{2}^{2}\leq \varepsilon_{K\text{-SVD}},\;\forall i, \end{array}$$

where $S=\left[\begin{array}{c} s_{\text{low}}\\ s_{\text{high}} \end{array}\right]=\left[s_{1},s_{2},\ldots ,s_{K}\right]$ stands for two sample sets that are one-to-one matching; $D=\left[\begin{array}{c} D_{l}\\ D_{h} \end{array}\right]$. It is worth noting that no more feature vectors are written in each dictionary atom except pixel values.

After updating the reconstruction result for each slice in the Fourier domain (restore the measured data), their work successfully reduce the PSNR of low-resolution MRI reconstruction images.

### 4.4. DDL in Multimodality Image Reconstruction

Multimodality biomedical imaging has found its increasing applications during the last decade and is becoming routine in clinical practice. Multimodality imaging is to integrate multiple imaging techniques into one instrument or fuse two or more imaging modalities such as CT, MRI, PET, and SPECT. This integration of structural, functional, and molecular information provides more accurate diagnoses. For example, MRI methods offer human soft tissue information with excellent clarity whereas CT depicts human hard tissue such as bone. Both of CT and MRI reveal important functional information. If these two modalities can be combined in one device, some small disease such as caducous blood clots could be exactly diagnosed. However, the imaging principles of MRI and CT are totally different, and how to build an accurate connection of these two modalities is an urgent problem.

**Figure 3:**
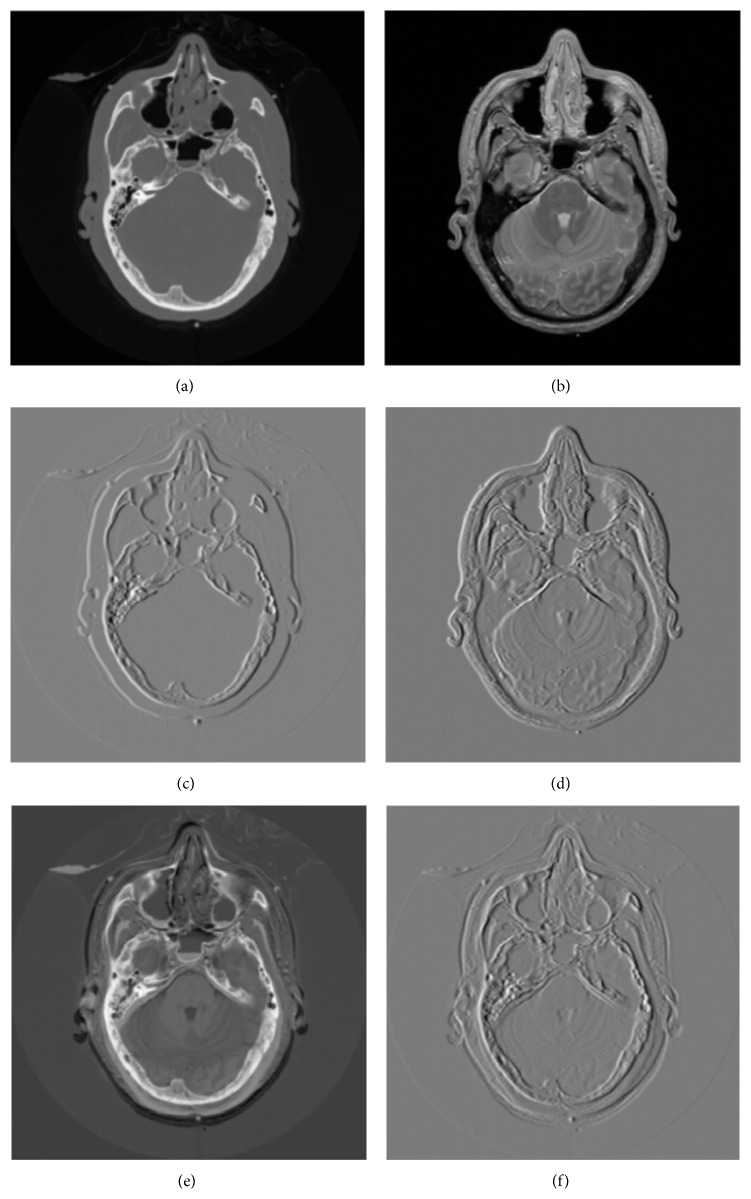
(a) CT image; (b) corresponding MRI image; (c) the first-order gradient of CT; (d) the first-order gradient of MRI; (e) CT and MRI images subtraction; (f) gradient images subtraction. (a) and (b) are obtained from* Visible Human Project* http://www.nlm.nih.gov/research/visible/visible_gallery.html.

In order to stylize the synergy between CT and MRI data sets from an object at the same time, Lu et al. try to investigate the possibility of CT-MRI unified imaging via dual-dictionary [46]. Figures 3(a) and 3(b) are, respectively, CT and MRI image; these two images are obtained from one layer of a patient's brain and are well registered. Figures 3(c) and 3(d) are the first-order gradient images of Figures 3(a) and 3(b) along *x* direction.  Figure 3(e) is the subtraction of CT and MRI, and Figure 3(f) is the subtraction of their gradients. From Figures 3(c), 3(d), and 3(f), we can see that the interiors of CT and MRI are structurally correlated, especially the brain bone. Thus, it is possible to build a connection of CT and MRI using the structural information. With an MRI image as the a priori information, Lu tries to recover its corresponding CT image.

Since CT scan is totally different with MRI scan in physical principle, they use direct method to reserve as much information as possible to establish a knowledge-based connection between the two datasets. The two dictionaries are *D*_MR_ and *D*_CT_; the former is derived from high-resolution MRI images, and the latter is from high-resolution CT images. The significant point of two dictionaries is that the patches in each dictionary are restricted one-to-one correspondence.

In reconstruction step, *D*_MR_ and *D*_CT_ are treated as *D*_*l*_ and *D*_*h*_ in (12), respectively. With dual-dictionary learning, a base CT image is first obtained just from a high-quality MRI image without corresponding CT data. Second, combined with base CT image and highly undersampled CT data, they reconstruct better resolution CT image using iterative method. The base CT image provides a better resolution and outline information, while highly undersampled CT image provides all the detailed information.

## 5. Discussion and Conclusion

In this paper, we discussed the recent advances of the DDL methods in medical imaging. Based on highly undersampled measured data, DDL algorithm has shown its great potential in reconstructing high-resolution images [47, 48].

Nowadays, MRI has become an indispensable medical modality of imaging diagnosis. However, during an MRI process, the scan time is usually up to fifteen minutes or even more. Patients might feel uncomfortable to keep motionless for a long time in the huge MRI gantry. Moreover, motion artifacts which reduce the images quality are always inevitable due to some organ movements such as heartbeat, pulse, and spasm. Researches demonstrated that the average displacement is over 0.35 mm within 100 seconds for one person lying on the cradle, while this number is up to 2.5 mm for a patient [42, 43]. Therefore, it has an important clinical significance to save the MRI scan time for better images quality and healthcare.

DDL method may be the future direction of fast MRI reconstruction. As mentioned in Section 4.4, the same slice of CT and MRI images from one object are structurally correlated. The advantage of CT is that the scanning time is short for some typical parts of body. Besides, the spatial resolution of CT is better than MRI. In the fast MRI, the measurement data is incomplete. Therefore, if the CT image data can be utilized as prior information in MRI reconstruction process, fewer measurement data (*k*-space) is required for high-resolution MRI image reconstruction. The essence of the reviewed DDL is establishing an appropriate relation between two spatial domains (e.g., different resolutions and different frequencies). One domain is for atom matching and the other domain is for image updating. Similarly, we may establish a quantitative relation between the two modalities using DDL. The relation can be a one-to-one mapping between the images boundaries which reflect the correlation between CT and MRI. In this way, DDL enables the fast MRI.

Overall, DDL method has shown its effective application in medical image reconstruction. With DDL method, we can reconstruct a high-resolution image with highly undersampling data. Inspired by its performances in one medical modality, DDL can be applied in structurally correlated image reconstruction problem, for example, multimodalities image reconstruction (CT-MRI).

However, the research work of DDL still remains in preliminary stage. For example, as discussed in the paper, reconstruction results may be relatively sensitive to the matching accuracy between the two dictionaries. Thus, how to establish closest connections between the images with different resolutions or even different modalities will be an important issue to be solved in the future. Also, the redundancy of dictionaries should be eliminated more reasonable to ensure better sparse representation.
